# Cavernous haemangioma and mid trimester pregnancy loss leading to severe haemorrhage and hysterectomy: a case report and review of literature

**DOI:** 10.52054/FVVO.15.4.111

**Published:** 2023-12-13

**Authors:** A Gallo, R D’Alisa, V Foreste, G Saccone, M.C. De Angelis, A Di Spiezio Sardo, B Zizolfi

**Affiliations:** Department of Public Health, University of Naples “Federico II”, Naples, 80131, Italy; Department of Maternal and Child Health and Urological Sciences, Sapienza University of Rome, Rome, 00185, Italy; Department of Neurosciences, Reproductive Sciences and Dentistry, University of Naples Federico II, Naples, 80131, Italy

**Keywords:** Haemangioma, pregnancy, bleeding, hysterectomy

## Abstract

**Background:**

Cavernous haemangiomas are benign vascular tumours that are known to occasionally involve the female genital tract, including the uterus. They are often underdiagnosed during pregnancy, although they can also lead to severe postpartum or antepartum haemorrhage.

**Objectives:**

Describe our case of an uncommon second-trimester pregnancy loss in a woman with a diffuse cavernous haemangioma of the uterus and cervix and review the wider literature.

**Methods:**

The review was conducted using MEDLINE, Scopus and PubMed electronic databases from beginning of the database to May 2023, using the following keywords: arteriovenous malformation; cavernous haemangioma/hemangioma; uterine neoplasms; pregnancy complications; abnormal vaginal bleeding.

**Main outcome measures:**

Description of the characteristics of cavernous haemangioma during pregnancy as well as diagnostic criteria and treatment options.

**Results:**

Twenty publications were included in the review, which included English-language case reports over a period from 1959 to 2022. No pathognomonic symptoms for cavernous haemangioma of the uterus in a pregnant woman were noted. Complications including massive secondary postpartum haemorrhage, haemoperitoneum, and severe thrombocytopenia with anaemia after delivery were reported.

**Conclusions:**

Diagnosis and management during pregnancy can be challenging and requires considerable attention, with a multidisciplinary approach including gynaecologists, radiologists, and pathologists to avoid major complications.

**What is new?:**

An additional case of diffuse cavernous haemangioma of the uterus and cervix is described, that adds to the little existing literature.

## Introduction

Cavernous haemangiomas are benign vascular tumours that rarely involve the female genital tract. They originate from either endothelial cells or pericytes that are found outside the vascular wall and consist of multiple anastomosing vessels lined by a single layer of endothelium. The appellative ‘cavernous’ comes from the observation that they consist of large anastomosing vascular spaces (‘caverns’), to differentiate from the ‘capillary’ haemangioma, a type of haemangioma composed of small size capillary vessels.

Fewer than 50 cases of uterine haemangioma have been reported so far, with 10 cases being during pregnancy (Benjamin et al., 2010; Aka et al., 2017). Uterine haemangiomas can be congenital or acquired.

The former appears to be associated with hereditary diseases, including hereditary haemorrhagic telangiectasia, tuberous sclerosis, blue rubber bleb nevus syndrome, Klippel-Trenaunay syndrome, Maffucci syndrome and Kasabach Merritt syndrome (Djunić et al., 2009; Shanberge, 1994; Verheijen et al., 1989; Jameson, 1990; Thanner et al., 2001; Virk et al., 2009). The latter is generally related to physical changes and hormonal alterations due to surgery, trophoblastic disease, pelvic inflammatory disease, endometrial carcinoma, or intake of diethylstilbestrol (Fleming et al., 1989; Frencken and Landman, 1965).

Usually, when haemangioma occurs on the uterine wall, the most involved portion is the myometrium, and sometimes the vessels may be spread throughout the uterus, cervix and fallopian tubes (Malhotra et al., 1995). While isolated cavernous haemangiomas are mostly found at the level of the myometrial layer, diffuse haemangiomas usually affect the entire uterine wall, thus extending from the endometrium to the serosa and, in some cases, as in this case, the cervix may be affected. Cases confirmed only to the cervix, even in pregnant women, have shown a good prognosis after both surgical removal and conservative approach.

Cavernous haemangioma of the uterus, whether isolated or diffuse, can be asymptomatic or lead to various complications, including heavy periods, intermenstrual spotting, infertility, and maternal and fetal death due to the severe bleeding in pregnant patients (Lotgering et al., 1989; Acess et al., 2020).

Although it can have serious consequences, especially during pregnancy, cavernous uterine haemangiomaa are often underdiagnosed. Indeed, diagnosis is very challenging not only because of the rarity of the condition but also because of the absence of any specific clinical features. Ultrasound (US) and magnetic resonance imaging (MRI) are used as imaging modalities to diagnose, however, the histopathological examination remains the gold standard diagnosis.

Regarding treatment, in cases of severe haemorrhage, when conservative management has failed, emergency hysterectomy may be necessary (Bhavsar et al., 2012).

In this manuscript, we present an uncommon case of second-trimester pregnancy loss in a woman with a cavernous haemangioma of the uterus. To better examine this data, a review of the published cases was also undertaken.

## Case Report

A 36-year-old nulliparous woman came to the emergency department of our institution (University of Naples Federico II, Naples, Italy) because of severe vaginal bleeding and abdominal pain during the 19th week of gestation.

**Figure 1:**
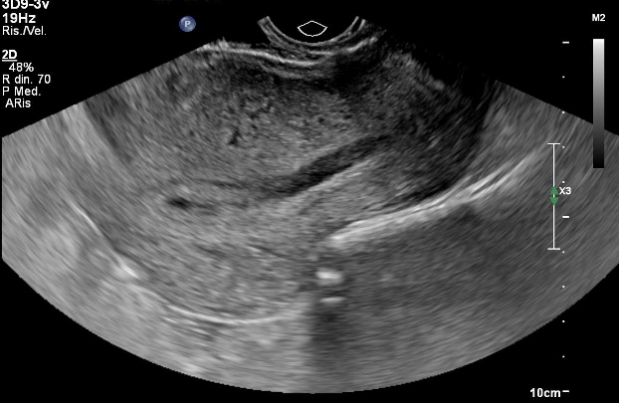
Ultrasound scan in the very early weeks of pregnancy, showing a thickened anterior wall with small anechogenic areas.

**Figure 2:**
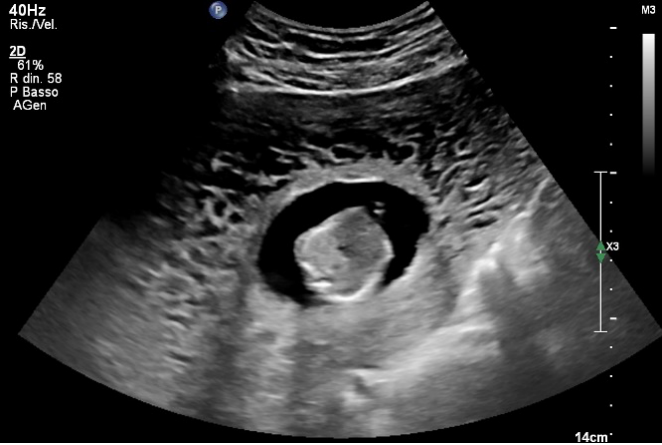
Ultrasound scan at 19 weeks of gestation showing diffusely thickened myometrium with anechogenic areas of larger size (Swiss-cheese appearance).

The patient’s surgical history reported a previous hysteroscopic metroplasty, performed in 2021 for a septate uterus. She had also had a previous first trimester miscarriage because of a blighted ovum and the current pregnancy was achieved by IVF. During the first trimester of pregnancy, there was US suspicion of an arteriovenous malformation (AVM) of the anterior wall of the uterus and cervix (Figures 1 and 2) and natural progesterone therapy was prescribed. US in the very early weeks of pregnancy, showed a thickened anterior wall with small anechogenic areas, while that performed at 19 weeks of gestation, showed a diffusely thickened myometrium with larger anechogenic areas (Swiss cheese appearance). However, when the patient presented to the emergency department a few weeks later, an office US was performed that showed a live fetus in a severely altered uterus, mainly due to increased size, symmetrically in both walls, with the presence of large anechogenic spaces within all myometrial walls and turbulent flow on power-Doppler. Despite prompt care to hemodynamically stabilise the patient, a few hours after admission, fetal heart rate (FHR) termination was found and consequently the pregnancy resulted in a second-trimester miscarriage. After the delivery, due to profuse bleeding, surgical evacuation with dilatation and curettage (D&C) under US guidance was performed. Bleeding persisted and intravenous Oxytocin 5 IU plus intramuscular Methylergometrine 500 mcg and finally Misoprostol 1 mg (5 tablets of 200 mcg) rectally were administered sequentially as uterotonic substances. Nevertheless, because of persistent severe bleeding, a haemostatic Bakri Balloon, filled with 150 cc of saline water, was inserted. Three hours after the procedure, the patient presented with a pale face and profuse sweating, with vital signs tending to instability. Vital parameters recorded were as follows: blood pressure 70/40 mmHg, heart rate 130 bpm, oxygen saturation 98%. Haemoglobin and haematocrit were 6 g/dl and 17,3% respectively (hemoglobin on admission: 10 g/dl). Two units of concentrated haematins and two units of plasma were transfused, in addition to intravenous infusion of electrolytes and tranexamic acid. Twenty-four hours after the procedure, the balloon was removed, and the persistence of severe vaginal bleeding was noted. US scan revealed a further increase in the size of the uterus, which reached the height of the umbilicus, with a sponge-like appearance, and several clots within the cavity.

**Figure 3:**
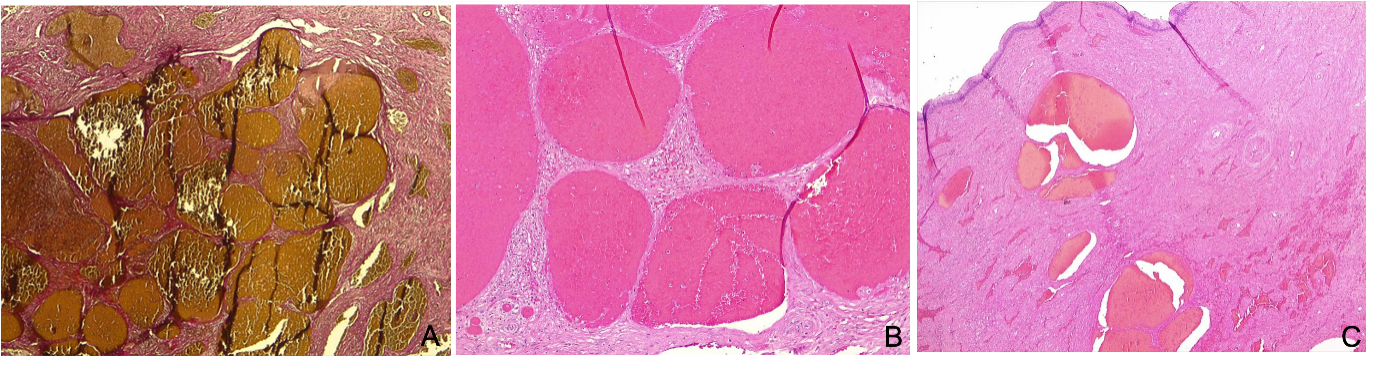
Microscopically detail of cavernous haemangioma. A. Negative elastic fibers stain to rule out the diagnosis of aterovenous malformation (AVM) (Van Gieson Stain). B-C: large thin-walled vessels in the wall of uterine (B) and cervix(C) (Hematoxylin-eosin).

**Figure 4:**
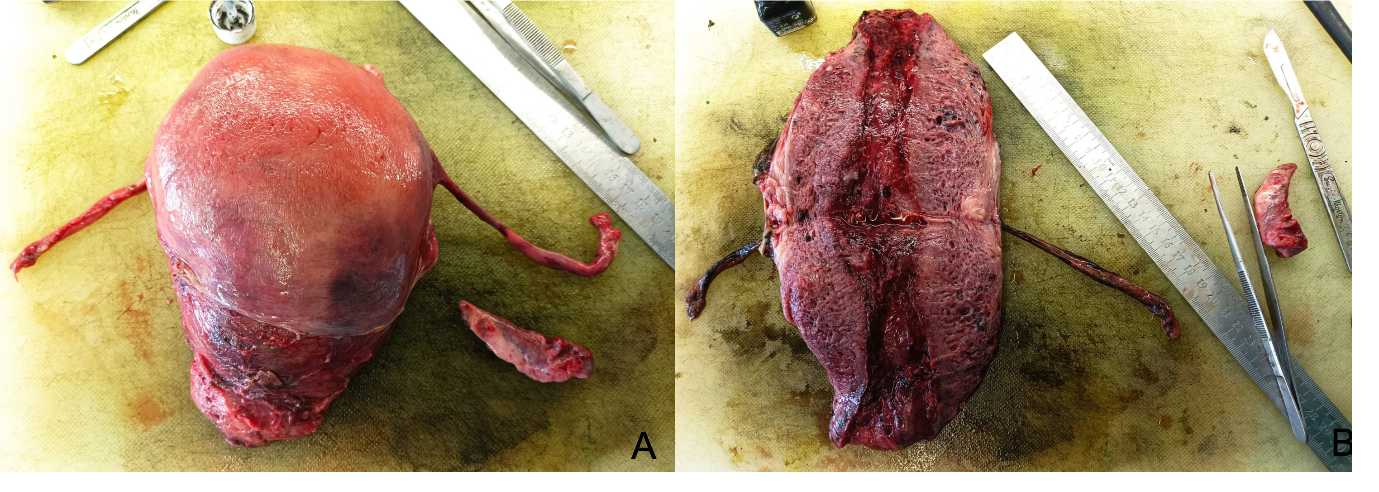
Anatomical detail of cavernous haemangioma. A. Macroscopic view of total hysterectomy specimen. B. Sectioned uterus; notice the thickened myometrium with a spongious appearance.

A CT scan confirmed increased uterine size and profuse bleeding from the uterine wall and cervix. On the basis of these investigations and the massive uncontrolled bleeding due to this type of diffuse cavernous haemangioma, an emergency total hysterectomy with prophylactic bilateral salpingectomy was then performed, promptly performing uterine artery ligation to control haemorrhage; the laparotomic approach was preferred in order to ensure rapid abdominal access and stabilise the patient. Histological examination confirmed the diagnosis of cavernous haemangioma (Figures 3-4), characterised by proliferation of thin-walled large vessels, and sporadic thrombosis of vascular structures. In order to exclude the diagnosis of AVM, an elastic fibers stain was performed, which was negative.

## Literature Review

### Methods

The systematic review was conducted using MEDLINE, Scopus and PubMed electronic databases from beginning of the database to May 2023, using the following keywords: arteriovenous malformation; cavernous haemangioma/ hemangioma; uterine neoplasms; pregnancy complications; abnormal vaginal bleeding. Additional studies were identified through other sources, such as manual searches through reference lists of articles we found before.

**Figure 5:**
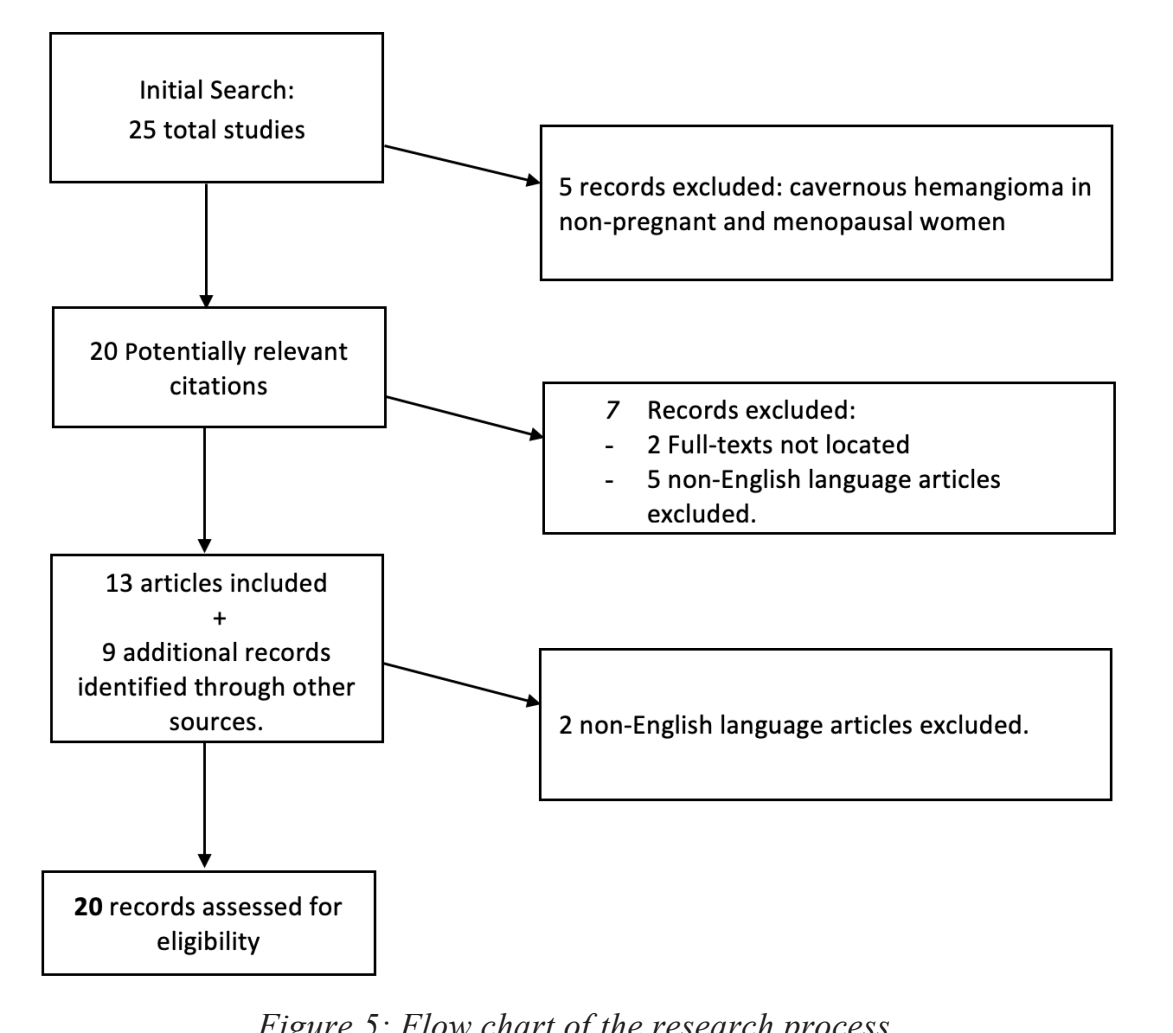
Flow chart of the research process.

Only cases of cavernous haemangioma in pregnant women were included in this review. Only English language papers were included. Studies published as abstracts only were excluded. Figure 5 illustrates the research process. Twenty items were included in the review (Table I), of which 12 concerned cavernous angiomas, four capillary angiomas, and one mixed angioma; in two articles this was not specified.

**Table I:** Summary of Reported Cases and review of uterine Haemangiomas during pregnancy and postpartum.

	Study location and year	Maternal characteristics (Age, parity, single or twin pregnancy)	Symptomatology, ultrasound findings, other	Gestational age	Pregnancy outcome (miscarriage, spontaneous or cesarean delivery)	Neonatal outcomes	Maternal outcomes	Histology
Lovett BF.	Philadelphia 1959	23 yo P0 Singleton pregnancy	Pelvic examination in the first trimester: 4 cm cervical lesion	At term	CS	Good general conditions	Good general conditions	Cavernous haemangioma of the cervix
Dawood MY, Teoh ES et al.	Singapore 1972	32 yo P0 Singleton pregnancy	No antenatal US findings Congenital haemangioma on left side of face and trunk Abdominal pain at 36w	36w	LSCS for failure to progress	FDIU	Hemoperitoneum (1500 mL) requiring transfusion	Numerous vascular channels consistent with haemangioma
Hadlock FP, Deter RL et al.	USA 1980	15 yo G2P1 (CS) Singleton pregnancy	Antenatal US: ‘snowflake’ pattern in myometrium Mild anemia	40w	LSCS	Good general conditions	Uterus preserved 1500 mL blood loss	Dilated mural venous channels in myometrium
Lotgering FK, Pijpers L et al.	Rotterdam 1989	32 yo P0 Singleton pregnancy	Enlarged uterus at 14w Premature rupture of the membranes and uterine contractions at 26w. Antenatal US: findings suggestive of uterine haemangioma	35w	Uncomplicated vaginal delivery with vacuum extraction and manual removal of placenta	NICU	Good general conditions	NS
Weissman A, Talmon R et al.	Israel 1993	NS P0 Singleton pregnancy	US at 30w: suspicion of incomplete molar pregnancy. Asymptomatic US at 33w: thickened myometrium with lacunar formations turbulent blood flow. Postpartum US: significant reduction in size of lesion	41w	Uncomplicated vaginal delivery	Good general conditions	Good general conditions	NS
Sutterlin MW, Müller T et al.	Germany 1998	30 yo G2P2 Singleton pregnancy	Abdominal pain US at 17w: no pathologic findings.	40w	CS	Good general conditions	Good general conditions	Uterus preserved. 600 mL blood loss Pregnancy induced vascular ectasia and edema
Thanner F, Suetterlin M et al.	Bern, Switzerland 2001	26 yo P0 Singleton pregnancy	Aneurysmal malformations in the lower body. LMWH since 10w for elevated D-Dimers US: diffusely thickened myometrium with numerous vascular channels. MRI diagnosis.	40w	LSCS	NICU for fetal bradycardia Severe vaginal bleeding post-partum with uterine atony and amniotic fluid embolism.	Uterus preserved. Massive secondary PPH at day 13 requiring transfusion	Cavernous haemangioma and angioleimyomatous hyperplasia
Riggs J, Bertoni M et al.	New York 2003	33 yo P0 Singleton pregnancy	Intractable cervical bleeding following pregnancy termination	At term	Vaginal delivery	Good general conditions	Bleeding persisted despite curettage and suturing, and ultimately required hysterectomy	Cavernous haemangioma
Comstock CH, Monticello ML et al.	USA 2005	NS G1P0 Singleton pregnancy	Antenatal US: diffusely thickened with numerous tubular echolucent areas.	40w	LSCS for failure to progress	Good general conditions	Uterus preserved. 1700 mL blood loss	Cavernous haemangioma
Virk RK, Zhong J et al.	USA 2009	21 yo G3-P2 (2 CS) Singleton pregnancy	No antenatal US findings. Mild anemia	40W	Elective CS	Good general conditions	Peripartum hysterectomy due to uncontrollable bleeding	Cavernous Haemangioma
Djunić I, Elezović IV et al.	Belgrado, Serbia 2009	33 yo P0 Singleton pregnancy	Congenital diffuse haemangioma of lower leg, gluteal region and labia. US uterine abnormalities at 24w Low grade DIC at 26w	28w	LSCS	FDIU	Uterus preserved.	Diffuse cavernous haemangioma with significant DIC
Guida M. Paladini D et al.	Italy 2009	20 yo P0 Singleton pregnancy	Swelling and lipotimic episodes. Vaginal, inguinal and vulvar varicosities. US: diffuse cavernous haemangiomatosis of the pregnant uterus	37w	CS	Good general conditions	Good general conditions	Diffuse cavernous haemangiomatosis
Benjamin MA, Yaakub HR et al.	Brunei, Asia 2010	27 yo P1 Singleton pregnancy	No antenatal US findings Asymptomatic	At term	Urgent LSCS for failure to progress	NS	Hysterectomy for profuse bleeding at 11 weeks post-partum	Diffuse ramifying haemangioma of the cervix and uterus with left hematosalpinx
Acevedo Gallegos S. Gallardo Gaona JM et al.	Spain 2011	NS P0 Singleton pregnancy	US at 16w: presumptive diagnosis of partial mole	38 w	CS	Not reported	Hysterectomy 24 hours after because of intra-abdominal haemorrhage	Cavernous haemangioma.
Bhavsar T, Wurzel J et al.	USA 2012	25 yo P0 Twin pregnancy	No antenatal US findings Asymptomatic	At term	LSCS	Good general conditions	Maternal death (pulmonary embolism and ARDS confirmed by lung autopsy)	Cavernous haemangioma (thrombosed cavernous haemangioma in the myometrium)
Mahapatra S, Das BP et al.	India 2012	27 yo P0 Singleton pregnancy	Pale, weak, and hypotensive, sensation of “something coming out” of her introitus. Speculum examination: 8 cm mass of the uterine cervix, bulging out through the introitus. US: hypoechoeic mass of 7 cm in the vagina, attached to the cervix	40w	Surgical resection of diagnosticated mass at 34w. CS	Good general conditions	Good general conditions	Cavernous haemangioma of the uterine cervix
Corbacioglu Esmer A, Özsürmeli M et al.	Istanbul, Turkey 2014	25 yo P0 Singleton pregnancy	Cutaneous haemangioma. Fetal growth restriction at 25w. US: diffusely thickened myometrium with numerous echolucent areas.	32w	LSCS	NICU (Fetal distress)	Uterus preserved. No significant post-partum bleeding.	Cavernous haemangioma
Knight P, Robertson M et al.	Australia 2016	28 yo G3P2 (2 CS) Singleton pregnancy	Anemia. USG suspected partial mole at 20w. MRI at 32w: thickened anterior myometrium with cystic spaces	35w	LSCS	Good general conditions	Good general conditions. Improvement in hemoglobin following delivery. No transfusion.	Cavernous haemangioma
Aka KE, Apollinaire Horo G et al.	Africa 2017	28 yo G3P2 Singleton pregnancy	No antenatal US findings. Asymptomatic.	40w	Uncomplicated vaginal delivery	Good general conditions	Severe thrombocytopenia and anemia postpartum. Blood and fresh plasma transfusion. Hysterectomy	Mixed histopathology: cavernous haemangioma of the body associated with a capillary haemangioma of the cervix
Bauters E, Aertsen M, Froyman W et al.	Leuven, Belgium 2022	30 yo P0 Singleton pregnancy	US at 9 w: overall diffusely thickened myometrium, with low flow rates in venous plexuses. MRI at 10 w: presumptive diagnosis of uterine haemangioma, due to marked T2-hyperintense, T1-hypotensive, non-diffusion restrictive enlargement of the entire myometrium enclosing multiple vessels.	39w	Vaginal delivery	Good general conditions	Postpartum haemorrhage with the placenta still in-utero. Manual removal of the placenta and curettage resolved uterus atony.	NS

Yo, years-old; G, gravida; P, para; LSCS, lower segment cesarean section; CS, cesarean section; PPH, postpartum haemorrhage; DIC, disseminated intravascular coagulation; FDIU, fetal death in utero; NICU: neonatal intensive care unit; MRI, magnetic resonance imaging; US, ultrasound; NS, not specified.

No pathognomonic symptoms for cavernous haemangioma of the uterus in a pregnant woman were identified. In the cases analysed, the symptomatology was characterised by vaginal bleeding, abdominal and pelvic pain, lipotimic episodes, anaemia, and altered coagulation factors; in one case, a diagnosis of congenital haemangiomas at other sites had been previously made. Massive secondary postpartum haemorrhage (PPH), haemoperitoneum, and severe thrombocytopenia with anaemia after delivery were reported in three articles. In these cases, maternal transfusions had to be performed to avoid fatal events (Aka et al., 2017; Thanner et al., 2001; Dawood et al., 1972).

A total of ten articles (Djunić et al., 2009; Thanner et al., 2001; Lotgering et al., 1989; Weissman et al., 1993; Sütterlin et al., 1998; Comstock et al., 2005; Hadlock et al., 1980; Guida et al., 2009; Çorbacıoğlu Esmer et al., 2014; Bauters et al., 2022) cited several prenatal US findings suggestive of uterine haemangioma, which we summarised in the Table II.

**Table II:** Summary of prenatal US findings suggestive of uterine haemangioma.

PRENATAL US FINDINGS SUGGESTIVE OF UTERINE HAEMANGIOMA
Diffusely thickened myometrium
Numerous echolucent areas with a Swiss-cheese appearance
Bidirectional flow at Color Doppler
Mixed arterial and venous pattern with low velocity at spectral Doppler
Severe fetal growth restriction
Early diastolic notch with a pulsatility index (PI) at Doppler examination of the bilateral uterine artery.

In three articles the prenatal US scan led to the diagnostic error of a cavernous haemangioma mistaken for a partial molar pregnancy (Knight et al., 2016; Weissman et al., 1993; Acevedo Gallegos et al., 2011); in two of these cases, histological examination on a biopsy specimen of the neoformation and on hysterectomy, revealed the diagnosis of cavernous haemangioma (Knight et al., 2016; Acevedo Gallegos et al., 2011). In two cases the diagnosis was confirmed by MRI (Thanner et al., 2001; Knight et al., 2016).

Good overall outcomes have been reported for both infants and mothers. In one case, after an uncomplicated vaginal delivery, US follow-up at 6 weeks after delivery demonstrated a significant reduction in the size of the lesion, thus discarding the hypothesis of a molar pregnancy (Weissman et al., 1993).

In seven cases, a diagnosis of cavernous haemangioma of the uterus was obtained only incidentally by histologic examination after hysterectomy, no clinical or ultrasonographic suspicion having been raised prenatally (Benjamin et al., 2010; Aka et al., 2017; Virk et al., 2009; Bhavsar et al., 2012; Sütterlin et al., 1998). In cases in which prenatal suspicion of cavernous haemangioma was raised by imaging (US, MRI) and caesarean section was performed without complications after extraction of the newborn, biopsy samples were taken of the visible neoformation (Djunić et al., 2009; Thanner et al., 2001; Lovett, 1959; Mahapatra et al., 2012; Sütterlin et al., 1998; Comstock et al., 2005; Hadlock et al., 1980; Guida et al., 2009; Çorbacıoğlu Esmer et al., 2014; Dawood et al., 1972).

In only one study, authors described a case of diffuse ramifying cavernous uterine haemangioma extending to the cervix, as in our case (Benjamin et al., 2010); in this case the neonatal outcome was good, but 11 weeks after the urgent caesarean delivery, the woman had to undergo a hysterectomy to control a heavy bleeding, after other measures had failed.

In two other articles cavernous haemangioma was confined to the cervix with the possibility of surgical resection or conservative management. In both cases, good pregnancy and delivery outcomes were reported (Lovett, 1959; Mahapatra et al., 2012). In one case, after vaginal delivery, the presence of a cervical haemangioma caused persistent bleeding despite curettage and suturing, necessitating total hysterectomy (Riggs et al., 2003). In all other cases analysed, haemangioma was present only on the uterine wall and in only one case was mixed, that is, both cavernous and capillary (Aka et al., 2017).

In asymptomatic cases, pregnancies resulted in term deliveries and lesions were found incidentally. In a few cases of mild abnormal uterine bleeding, pregnancy was monitored more closely with haematochemical tests and US scans every fortnight.

Five articles reported cases of preterm deliveries; among them, three of five preterm infants were born alive. One of them presented with a good Apgar score at birth (9/9) (Knight et al., 2016). Three preterm infants were admitted to the Neonatal Intensive Care Unit (NICU), due to Preterm Premature Rupture of Membranes (pPROM) with preterm vaginal delivery with vacuum extraction (Lotgering et al., 1989), fetal distress after preterm cesarean delivery at 32 weeks of gestation (Çorbacıoğlu Esmer et al., 2014), and fetal bradycardia after term caesarean delivery (Thanner et al., 2001), respectively. In these pregnancies, US suspicion of haemangioma arose during the second trimester and uterine contractions and uterine bleeding occurred in the latter two cases. Two cases of intrauterine fetal death (IUFD) were described, at 28 and 36 weeks, respectively (Djunić et al., 2009).

Twin pregnancies with an inauspicious outcome were described in two articles. In the first case, a vaginal delivery at term was followed by neonatal and maternal death (Kaufmann, 1997). In the second case, there was a caesarean delivery followed by hysterectomy due to uncontrollable haemorrhage. Maternal death occurred one week after delivery from pulmonary embolism and acute respiratory distress syndrome (ARDS), while the general condition of the infant was good (Bhavsar et al., 2012).

## Discussion

Cavernous haemangiomas are benign vascular neoplasms that can occasionally affect the uterus. It does not have a prevalence at any age, so it can affect women of all ages (Malhotra, 1995).

The aetiology of uterine cavernous haemangiomas has not been fully elucidated. However, it appears that uterine haemangioma cells may originate as pluripotent, embryogenic, mesodermal cells in the uterus (Chou and Chang, 2012). A pathogenetic theory on acquired haemangioma proposed by Sun et al. (2008) states that stimulation of oestrogen hormones could activate angiogenic factors such as Vascular Endothelial Growth Factor (VEGF) that would fuel the growth of the lesion. In these cases, haemangiomas may also present as endometrial polyps (Sharma et al., 2006).

The haemangioma may be a target tissue for oestrogen, and this would explain why the lesion may increase in size or regress during pregnancy and the postpartum period, respectively. Further studies are needed to precisely understand the mechanisms of oestrogen-induced haemangioma formation (Reggiani Bonetti et al., 2009).

Conditions that can be mistaken for haemangiomas include molar pregnancy or AV malformation or angiomas and hemangioendotheliomas, which can only be identified by histologic examination. If performed properly, prenatal US scans may show the presence of a thickened uterine lining and a mixed echo pattern suggesting cavernous changes with slow turbulent flow. Color Doppler and MRI can be very helpful in identifying this medical condition. Arteriographies and CT scans can also be helpful but are not recommended during pregnancy. However, histological examination is still considered the gold standard for the diagnosis of cavernous haemangiomas, as several cases are classified as cavernous haemangiomas of the uterus only after histological examination. (Lotgering et al., 1989)

Few cases of pregnancy-related uterine haemangiomas are reported in the literature (Aka et al., 2017). Clinical presentation ranges from asymptomatic (when the lesion is small) to anaemia, abdominal pain, excessive vaginal bleeding, infertility, and recurrent miscarriages. When confined only to the cervix it seems to have better prognosis in terms of management and treatment. In pregnant women, serious pregnancy-related complications may occur, even in the postpartum period, and a close follow-up is necessary to prevent short-term complications, such as amniotic fluid embolism, and long-term complications, such as disseminated intravascular coagulation (DIC), uterine atony, and thromboembolism or acute respiratory distress syndrome. DIC may be triggered by hormonal and physiological changes due to pregnancy (e.g., increased blood volume and others) that could make pre-existing vascular lesions manifest (Djunić et al., 2009). US follow-up sometimes shows a complete disappearance of the lesions 6 to 12 months after delivery, but unfortunately, it cannot be determined whether this regression is due to the lack of postpartum hormonal stimulation or the natural history of haemangiomas (Virk et al., 2009).

It is not yet clear which is the best way to treat haemangiomas; the management’s goal is recognition of the underlying condition and potential for severe complications, proper preparation, including consultant obstetrician and anesthetic involvement.

Delivery is preferred by conservative methods, but if caesarean section is necessary, the procedure should be performed under general anesthesia, and packaged units of plasma and red blood cells should always be immediately available.

When diagnosed, conservative treatment should be performed in case of haemorrhage, through various therapeutic approaches (i.e., uterine embolisation or internal artery ligation, electrocautery, radiotherapy, cryotherapy, carbon dioxide laser excision, and knife excision). The results obtained are still debated because most of them cannot prevent complications during pregnancy (Poppe et al., 1987). Selective embolisation of uterine arteries is very useful in cases of active massive haemorrhage and involves injection of an embolising agent through a catheter to obstruct the abnormal blood vessels of the angioma and thus reduce its size. It was not considered in our case, within the observation window to try to avoid a hysterectomy, because it is not available at our institution. The angioma can also be completely removed by laparoscopic excision, especially when it is small and confined to a part of the uterus; therefore, the choice of this procedure depends on the location and severity of the angioma. In cases of early pregnancy loss, hysteroscopy can be used for diagnosis and follow-up of arteriovenous malformations when ultrasound is inconclusive. It can therefore be critical to prevent haemorrhage due to inappropriate curettage. In fact, dilatation and curettage (D&C) procedure should be avoided in these patients because it can worsen blood loss due to rupture of the congested vessels. Therefore, hysterectomy may be necessary in these patients to bring life-threatening haemorrhage under control. (Fleming, 1989; McLachlan et al., 1978; Pedowitz et al., 1955; Bauters et al., 2022).

In our experience, a 20-week miscarriage was observed in a patient with multiple uterine and cervical cavernous haemangiomas. The main limit of our management was the fact that the diffuse branching haemangioma of the cervix and of the uterus was not clearly identified by US examination at the time of our patient’s initial admission. Underdiagnosis may have led to a delay in treatment that may have prevented the uterine loss.

## Conclusions

Pregnancy in a patient with diffuse cavernous haemangiomas of the uterus and cervix is extremely rare; this condition may be clinically silent during pregnancy and delivery, or it may mimic other conditions causing severe vaginal bleeding. Establishing the diagnosis of uterine haemangioma before delivery is mandatory, as the complications associated with uterine haemangioma in pregnancy are considerable. Prenatal diagnosis can be extremely challenging, but it can lead to better outcomes by giving the patient the possibility to be managed in a multidisciplinary approach with gynecologists, radiologists, and anesthesiologists to avoid life-threatening complications or hysterectomy. Prenatal US, from what emerges from the literature, may be the first-line approach, followed by MRI; these techniques may arouse suspicion, but there are no standardised criteria for making a definite diagnosis. In the definitive diagnosis and differentiation between categories of angiomas, anatomopathologists also play a crucial role. It would be necessary to define diagnostic criteria and improve US techniques to detect signs of pathology as early as possible. For treatment, interventional radiology techniques (i.e. embolisation) should be considered to avoid abnormal bleeding and carry on the pregnancy, or if demolitive surgery is necessary, minimally invasive surgical techniques (i.e. laparoscopic excision) should be considered.
